# Family Physicians’ Approaches to Mental Health Care and Collaboration with Psychiatrists

**DOI:** 10.7759/cureus.4755

**Published:** 2019-05-25

**Authors:** Norah Althubaiti, Ranya Ghamri

**Affiliations:** 1 Obstetrics and Gynecology, King Abdulaziz University, Jeddah, SAU; 2 Family Medicine, King Abdulaziz University, Jeddah, SAU

**Keywords:** family physicians, psychiatry, mental health, psychiatrists, primary health care

## Abstract

Objective

This study aimed to determine the proportion of family physicians referring patients to psychiatrists and conducting psychotherapy or mental health consultations themselves. Additionally, the factors affecting family physicians’ approaches to dealing with mental health patients were investigated, including referrals to psychiatrists and physicians’ views about better management plans for patients with mental health disorders.

Method

In this cross-sectional observational study, online surveys were distributed, using Google forms, to family physicians in primary healthcare centers and hospitals in Jeddah, Saudi Arabia. The participants were 175 family physicians. A previously developed survey under the name "collaboration between psychologists and primary health care physicians" was adapted to suit the purposes of the present study, by changing the aim of the survey from psychologists to family physicians.

Results

Physicians who received inter-professional training in a clinical training program were more likely to agree that their education prepared them well for collaboration with psychiatrists, compared to those who did not receive such an education (p<0.001). The younger and less experienced physicians were more likely to carry out psychotherapy and mental health consultations by themselves more often than were the more experienced physicians (33.1% versus 9.7%; p<0.001), it has also been shown that almost 90% of physicians agreed that collaboration with psychiatrists is necessary for the care of their patients, and only a third responded that psychiatrists were accessible if and when they want to consult with them.

Conclusions

Family and primary care physicians must collaborate with psychiatric professionals in order to provide effective services. Moreover, family physicians should receive more education about mental health, and effective communication should be encouraged in order to deliver better care to psychiatric patients in primary healthcare settings.

## Introduction

The importance of primary healthcare in improving health outcomes and managing costs has been recognized worldwide; however, there is still room for improvement in many parts of the world [1]. Mental health problems are often encountered in primary healthcare centers, and family physicians play an important role in caring for mentally ill patients [2]. One study conducted in Canada showed that 40% of patients presenting with mental health problems were only seen by their family physicians [3]. In fact, a study that was done in central Saudi Arabia showed that 18% of primary care patients had mental health issues [4]. While another study showed an even higher percentage of 26.5% [5]. More importantly, family physicians are often the first point of contact for these patients [6].

According to the World Health Organization (WHO), “integrating mental health services into primary care is the most viable way of closing the treatment gap and ensuring that people get the mental health care they need" [7]. Thus, it is important to make sure that family physicians are confident in their approach to treating mental health disorders. When family physicians were surveyed in Canada, fewer than half (46%) reported being satisfied with the mental health care they were able to deliver to their patients [2]. However, a much higher level of satisfaction was found when there was a mental health practitioner on site [2]. Most of the family physicians also stated that access to mental health practitioners was a critical element for improving the delivery of mental health services in a primary care setting [2]. Therefore, it is important that mental health patients are provided with adequate care when visiting their family physicians. This study aimed to contribute to that objective by surveying family physicians in primary healthcare centers and hospitals in Jeddah, Saudi Arabia, about the care that they provide for their mental health patients, the factors affecting it, and what they believe could improve it.

## Materials and methods

Participants and measures

This cross-sectional observational study was carried out between January 2017 and January 2018 in primary healthcare centers and hospitals in Jeddah, Saudi Arabia. Family physicians participated in this study, including consultants and residents at all residency levels, from both primary healthcare centers and hospitals in all sectors. An online survey was distributed among them using Google Forms. The aims of this research and a complete description of the procedures were explained in the survey, and all participants were required to sign an electronic informed consent form before participating. We used a survey previously developed by Drewlo [8], which we adjusted to suit our research questions. The survey consisted of 22 questions, including demographic data and the frequency with which the participating physicians had referred or treated a mental health patient during the previous year. The survey also asked questions about the participants’ confidence in treating a mental health patient (e.g., detecting and treating an issue or collaborating with psychiatrists), availability of psychiatrists for referral, opinions about what could improve the mental health care provided to patients, and factors affecting such care. Ethical approval was obtained from the King Abdulaziz University Hospital ethics committee (approval number 366-17) and the appropriate procedures were followed in accordance with the ethical standards of the committee.

Statistical analysis

The data from the questionnaires were analyzed using the Statistical Package for the Social Sciences (SPSS Inc., Chicago, IL, USA). The statistical analysis of the data included the number and frequency characteristics (%). Pearson’s chi-square or Fisher’s exact tests were used to determine whether there were significant differences between the expected frequencies (e.g., categorical variables). A p-value of less than 0.05 was considered statistically significant.

## Results

Family physicians’ attitudes toward collaboration with psychiatrists

This study included 175 physicians, and their characteristics are summarized in Table 1. Over half (54.3%) were between 25 and 34 years old, and 21.7% were 45 or older. Women represented 60.6% of the respondents. Approximately one-third of the physicians (33.1%) had less than five years of experience while 12.6% had 20 or more. Nearly half (43.4%) worked at primary healthcare centers, and 10.9% worked in academic settings. Most of the participants (70.3%) had received training in mental illnesses, and 45.7% had interned in psychiatric settings. Additionally, most of the physicians (74.3%) received inter-professional education as part of their doctoral program, and 72% received inter-professional training in a clinical training program. A total of 73 physicians (41.7%) reported referring patients with mental health disorders to a psychiatrist or consulted a psychiatrist about patients with mental health disorders monthly, whereas 30.9% did this once a year or less and 5.1% never did so. Moreover, 20% of the physicians reported conducting psychotherapy and mental health consultations themselves while 18.3% never did so.

**Table 1:** Physicians’ demographic and work characteristics (N=175)

Characteristics	N	%
Age (years)		
25–34	95	54.3
35–44	42	24
45 and older	38	21.7
Gender		
Male	69	39.4
Female	106	60.6
Years of practice		
0–4	58	33.1
5–9	45	25.7
10–14	33	18.9
15–20	17	9.7
More than 20	22	12.6
Work setting		
Academic	19	10.9
Primary healthcare center	76	43.4
Hospital	32	18.3
Mixed (working in different settings)	48	27.4
Received training in mental health		
Yes	123	70.3
No	50	28.6
Other	2	1.1
Interned in a psychiatric setting		
Yes	80	45.7
No	95	54.3
Received inter-professional education as part of a doctoral program		
Yes	130	74.3
No	45	25.7
Received inter-professional training in a clinical training program		
Yes	126	72.0
No	49	28.0
Referral and consultation with psychiatrists for mental health patients		
More than once a day	1	0.6
Once a day	11	6.3
Weekly	17	9.7
Biweekly	10	5.7
Monthly	73	41.7
Once a year or less	54	30.9
Never	9	5.1
Carries out psychotherapy and mental health consultations him/herself		
More than once a day	11	6.3
Once a day	23	13.1
Weekly	33	18.9
Biweekly	16	9.1
Monthly	35	20.0
Once a year or less	25	14.3
Never	32	18.3

Comparison of attitudes by family physician’s gender

As illustrated in Table 2, more male physicians (73.9% versus 56.5%) agreed that their education prepared them well for collaboration with psychiatrists (p=0.02). Similarly, more male physicians (59.4% versus 38.7%) agreed that their current collaboration with psychiatrists helped them optimize their client care (p=0.007). However, more female physicians (81.1% versus 63.8%) believed that mental health physicians should be employed in primary healthcare settings (p=0.010). That is explained as only one-third of both genders has access to psychiatrists (p=0.096). There are no significant differences between males and females; in response to that, family physicians need to be better educated and trained regarding the identification of mental health problems. The majority of both genders agreed that they need to be more trained (p=0.784). There was no significance in the difference between age groups in the comparison of attitudes.

**Table 2:** Physicians’ agreement on collaboration with psychiatrists by gender

Items	Total (n=175) n (%)	Gender n (%)		p-value
		Male (n=69)	Female (n=106)	
My education prepared me well for collaboration with psychiatrists.	111 (63.4)	51 (73.9)	60 (56.6)	0.020
I think family physicians need to be better educated and trained regarding the identification of mental health problems.	161 (92.0)	63 (91.3)	98 (92.5)	0.784
My current collaboration with psychiatrists is effective for optimizing client care.	82 (46.9)	41 (59.4)	41 (38.7)	0.007
I am comfortable giving feedback about a client to their psychiatrist.	124 (70.9)	54 (78.3)	70 (66.0)	0.082
There is a hierarchy in my relationship with the mental health physicians with whom I work.	62 (35.4)	24 (34.8)	38 (35.8)	0.885
Psychiatrists are accessible if and when I want to consult with them.	63 (36.0)	30 (47.6)	33 (52.4)	0.096
I am accessible if psychiatrists want to consult with me.	122 (69.7)	49 (71.0)	73 (68.9)	0.763
I think my collaboration with my client’s psychiatrist is necessary for the care of my client.	158 (90.3)	59 (85.5)	99 (93.4)	0.085
I feel respected by psychiatrists during our periods of contact regarding patient care.	104 (59.4)	42 (60.9)	62 (58.5)	0.754
I think that mental health physicians should be employed in primary healthcare settings.	130 (74.3)	44 (63.8)	86 (81.1)	0.010

Comparison of attitudes by work setting

As shown in Table 3, physicians working in hospital-based settings were more likely to agree that psychiatrists were accessible if and when they wanted to consult with them (62.5%) as compared to physicians working in academic, primary health care centers, and mixed settings (36.8%, 34.2%, 20.8%, respectively; p=0.002).

**Table 3:** Physicians’ agreement on collaboration with psychiatrists based on work setting

Items	Total (n=175) n (%)	Work Setting n (%)				p-value
		Academic (n= 19)	Primary Healthcare Centers (n=76)	Hospital (n=32)	Mixed (n=48)	
My education prepared me well for collaboration with psychiatrists.	111 (63.4)	8 (42.1)	54 (71.1)	24 (75.0)	25 (52.1)	0.172
I think family physicians need to be better educated and trained regarding the identification of mental health problems.	161 (92.0)	18 (94.7)	69 (90.8)	27 (84.4)	47 (97.9)	0.161
My current collaboration with psychiatrists is effective for optimizing client care.	82 (46.9)	8 (42.1)	36 (47.4)	18 (56.3)	20 (41.7)	0.608
I am comfortable giving feedback about a client to their psychiatrist.	124 (70.9)	13 (68.4)	56 (73.7)	24 (75.0)	31 (64.6)	0.675
There is a hierarchy in my relationship with the mental health physicians with whom I work.	62 (35.4)	4 (21.1)	32 (42.1)	11 (34.4)	15 (31.3)	0.311
Psychiatrists are accessible if and when I want to consult with them.	63 (36.0)	7 (36.8)	26 (34.2)	20 (62.5)	10 (20.8)	0.002
I am accessible if psychiatrists want to consult with me.	122 (69.7)	14 (73.7)	49 (64.5)	27 (84.4)	32 (66.7)	0.204
I think my collaboration with my client’s psychiatrist is necessary for the care of my client.	158 (90.3)	17 (89.5)	70 (92.1)	28 (87.5)	43 (89.6)	0.894
I feel respected by psychiatrists during our periods of contact regarding patient care.	104 (59.4)	9 (47.4)	49 (64.5)	21 (65.6)	25 (52.1)	0.317
I think that mental health physicians should be employed in primary healthcare settings.	130 (74.3)	15 (78.9)	60 (78.9)	19 (59.4)	36 (75.0)	0.186

Comparison of attitudes by mental health training

More physicians who received training in mental health issues agreed that their education prepared them well for collaboration with psychiatrists as compared to those who did not receive such training (71.5% versus 44.2%, respectively; p=0.001; Table 4).

**Table 4:** Physicians’ agreement on collaboration with psychiatrists based on mental health training

Items	Total (n=175) n (%)	Training in Mental Health n (%)		p-value
		Yes (n=123)	No (n=52)	
My education prepared me well for collaboration with psychiatrists.	111 (63.4)	88 (71.5)	23 (44.2)	0.001
I think family physicians need to be better educated and trained regarding the identification of mental health problems.	161 (92.0)	115 (93.5)	46 (88.5)	0.262
My current collaboration with psychiatrists is effective for optimizing client care.	82 (46.9)	63 (51.2)	19 (36.5)	0.075
I am comfortable giving feedback about a client to their psychiatrist.	124 (70.9)	87 (70.7)	37 (71.2)	0.955
There is a hierarchy in my relationship with the mental health physicians with whom I work.	62 (35.4)	42 (34.1)	20 (38.5)	0.585
Psychiatrists are accessible if and when I want to consult with them.	63 (36.0)	46 (37.4)	17 (32.7)	0.553
I am accessible if psychiatrists want to consult with me.	122 (69.7)	85 (69.1)	37 (71.2)	0.788
I think my collaboration with my client’s psychiatrist is necessary for the care of my client.	158 (90.3)	114 (92.7)	44 (84.6)	0.100
I feel respected by psychiatrists during periods of contact regarding patient care.	104 (59.4)	77 (62.6)	27 (51.9)	0.189
I think that mental health physicians should be employed in primary healthcare settings.	130 (74.3)	87 (70.7)	43 (82.7)	0.098

Comparison of attitudes by internship in mental health

There was no significant association between agreement with the statement concerning collaboration with psychiatrists and having had an internship in mental health (see Appendix).

Comparison of attitudes by inter-professional education

Table 5 shows that more physicians who received inter-professional education as part of their doctoral program agreed that their education prepared them well for collaboration with psychiatrists as compared to those who did not receive such an education (71.5% versus 40%; p<0.001).

**Table 5:** Physician’s agreement on collaboration with psychiatrists based on inter-professional education in the doctoral program

Items	Total (n=175) n (%)	Received Inter-Professional Education n (%)		p-value
		Yes (n=130)	No (n=45)	
My education prepared me well for collaboration with psychiatrists.	111 (63.4)	93 (71.5)	18 (40.0)	<0.001
I think family physicians need to be better educated and trained regarding the identification of mental health problems.	161 (92.0)	120 (92.3)	41 (91.1)	0.799
My current collaboration with psychiatrists is effective for optimizing client care.	82 (46.9)	65 (50.0)	17 (37.8)	0.157
I am comfortable giving feedback about a client to their psychiatrist.	124 (70.9)	94 (72.3)	30 (66.7)	0.473
There is a hierarchy in my relationship with the mental health physicians with whom I work.	62 (35.4)	45 (34.6)	17 (37.8)	0.702
Psychiatrists are accessible if and when I want to consult with them.	63 (36.0)	51 (39.2)	12 (26.7)	0.130
I am accessible if psychiatrists want to consult with me.	122 (69.7)	94 (72.3)	28 (62.2)	0.204
I think my collaboration with my client’s psychiatrist is necessary for the care of my client.	158 (90.3)	118 (90.8)	40 (88.9)	0.714
I feel respected by psychiatrists during our periods of contact regarding patient care.	104 (59.4)	81 (62.3)	23 (51.1)	0.187
I think that mental health physicians should be employed in primary healthcare settings.	130 (74.3)	98 (75.4)	32 (71.1)	0.572

Comparison of attitudes by inter-professional training in a clinical training program

Table 6 shows that the physicians who received inter-professional training in a clinical training program agreed that their education prepared them well for collaboration with psychiatrists as compared to those who did not receive such an education (71.4% versus 42.9%, respectively, p<0.001).

**Table 6:** Physicians’ agreement on collaboration with psychiatrists based on inter-professional training in a clinical training program

				p-value
Items	Total (n=175) n (%)	Received Inter-Professional Training n (%)		
		Yes (n=126)	No (n=49)	
My education prepared me well for collaboration with psychiatrists.	111 (63.4)	90 (71.4)	21 (42.9)	<0.001
I think family physicians need to be better educated and trained regarding the identification of mental health problems.	161 (92.0)	118 (93.7)	43 (87.8)	0.197
My current collaboration with psychiatrists is effective for optimizing client care.	82 (46.9)	64 (50.8)	18 (36.7)	0.094
I am comfortable giving feedback about a client to their psychiatrist.	124 (70.9)	92 (73.0)	32 (65.3)	0.314
There is a hierarchy in my relationship with the mental health physicians with whom I work.	62 (35.4)	43 (34.1)	19 (38.8)	0.564
Psychiatrists are accessible if and when I want to consult with them.	63 (36.0)	44 (34.9)	19 (38.8)	0.633
I am accessible if psychiatrists want to consult with me.	122 (69.7)	91 (72.2)	31 (63.3)	0.247
I think my collaboration with my client’s psychiatrist is necessary for the care of my client.	158 (90.3)	114 (90.5)	44 (89.8)	0.891
I feel respected by psychiatrists during our periods of contact regarding patient care.	104 (59.4)	77 (61.1)	27 (55.1)	0.467
I think that mental health physicians should be employed in primary healthcare settings.	130 (74.3)	92 (73.0)	38 (77.6)	0.538

Factors associated with conducting psychotherapy and mental health consultations

Younger physicians (25-34 years) were more likely to carry out psychotherapy and mental health consultations by themselves more often than were older physicians (≥45 years; 44.8% versus 22.4%, respectively). However, this difference was almost but not quite significant (p=0.052). Similarly, the less experienced physicians (0-4 years) were more likely to carry out psychotherapy and mental health consultations by themselves more often than were more experienced physicians (15-20 years; 22.4% versus 9.0%, respectively), and this difference was significant (p<0.001).

Moreover, physicians who had received training in mental health were significantly more likely to carry out psychotherapy and mental health consultations by themselves more often than were those without training (80.6% versus 19.4%, respectively; p=0.003). Additionally, those who received inter-professional education as part of their doctoral program, which is taking a psychiatry rotation within the doctoral program, were significantly more likely to carry out psychotherapy and mental health consultations by themselves more often than were those without such education (83.6% versus 16.4%, respectively; p=0.001). Similarly, physicians who received inter-professional training in a clinical training program were significantly more likely to carry out psychotherapy and mental health consultations by themselves more often than were those without training (79.1% versus 20.9%, respectively; p=0.017).

Physicians who agreed with the following statements were more likely to carry out psychotherapy and mental health consultations by themselves more often than were those who disagreed with these statements: their education prepared them well for collaboration with psychiatrists (p<0.001), family physicians need to be better educated and trained regarding the identification of mental health problems (p=0.011), they are comfortable giving feedback about a client to their psychiatrist (p=0.044), and they feel respected by psychiatrists when contacting them regarding patient care (p=0.003; Table 7).

**Table 7:** Factors associated with physicians conducting psychotherapy and mental health consultations

Items		Total (n=175) n (%)	Conducting Psychotherapy and Mental Health Consultations				
			Very often n (%) (n= 67)	Sometimes n (%) (n=51)	Rarely (n=25) n (%)	Never (n=32) n (%)	p-value
Gender	Male	69 (39.4)	23 (34.4)	22 (43.1)	11 (44.0)	13 (40.6)	0.738
	Female	106 (60.6)	44 (65.7)	29 (56.9)	14 (56.0)	19 (59.4)	
Age group (years)	25–34	95 (54.3)	30 (44.8)	25 (49.0)	18 (72.0)	22 (68.8)	0.052
	35–44	42 (24.0)	22 (32.8)	13 (25.5)	1 (4.0)	6 (18.8)	
	45 and older	38 (21.7)	15 (22.4)	13 (25.5)	6 (24.0)	4 (12.5)	
Years of practice	0–4	58 (33.1)	15 (22.4)	12 (23.5)	12 (48)	19 (59.4)	<0.001
	5–9	45 (25.7)	15 (29.4)	6 (28.0)	16 (18.8)	17 (25.4)	
	10–14	33 (18.9)	21 (31.3)	9 (17.6)	0 (0.0)	3 (9.4)	
	15–20	17 (9.7)	6 (9.0)	10 (19.6)	1 (4.0)	0 (0.0)	
	More than 20	22 (12.6)	8 (11.9)	5 (9.8)	5 (20.0)	4 (12.5)	
Work setting	Academic	19 (10.9)	5 (7.5)	3 (5.9)	5 (20.0)	6 (18.8)	0.094
	Primary Healthcare Center	76 (43.4)	37 (55.2)	22 (43.1)	8 (32.0)	9 (28.1)	
	Hospital	32 (18.3)	9 (13.4)	11 (21.6)	7 (28.0)	5 (15.6)	
	Mixed	48 (27.4)	16 (23.9)	15 (29.4)	5 (20.0)	12 (37.5)	
Received training in mental health issues	Yes	123 (70.3)	54 (80.6)	39 (76.5)	15 (60.0)	15 (46.9)	0.003
	No	52 (29.7)	13 (19.4)	12 (23.5)	10 (40.0)	17 (53.1)	
Interned in a psychiatric setting	Yes	80 (45.7)	28 (41.8)	22 (43.1)	11 (44.0)	19 (59.4)	0.393
	No	95 (54.3)	39 (58.2)	29 (56.9)	14 (56.01)	13 (40.6)	
Received inter-professional education	Yes	130 (74.3)	56 (83.6)	39 (76.5)	20 (80.0)	15 (46.9)	0.001
	No	45 (25.7)	11 (16.4)	12 (23.5)	5 (20.0)	17 (53.1)	
Received inter-professional training	Yes	126 (72.0)	53 (79.1)	33 (64.7)	22 (88.0)	18 (56.3)	0.017
	No	49 (28.0)	14 (20.9)	18 (35.3)	3 (12.0)	14 (43.8)	
My education prepared me well for collaboration with psychiatrists.	Agree	111 (63.4)	55 (82.1)	32 (62.7)	12 (48.0)	12 (37.5)	<0.001
	Disagree	64 (36.6)	12 (17.9)	19 (37.3)	13 (52.0)	20 (62.5)	
Family physicians need to be better educated and trained regarding the identification of mental health problems.	Agree	161 (92.0)	64 (95.5)	49 (96.1)	19 (76.0)	29 (90.6)	0.011
	Disagree	14 (8.0)	3 (4.5)	2 (3.9)	6 (24.0)	3 (9.4)	
My current collaboration with psychiatrists is effective for optimizing client care.	Agree	82 (46.9)	35 (52.2)	23 (45.1)	15 (60.0)	9 (28.1)	0.069
	Disagree	93 (53.1)	32 (47.8)	28 (54.9)	10 (40.0)	23 (71.9)	
I am comfortable giving feedback about a client to their psychiatrist.	Agree	124 (70.9)	55 (82.1)	35 (68.6)	16 (64.0)	18 (56.3)	0.044
	Disagree	51 (29.1)	12 (17.9)	16 (31.4)	9 (36.0)	14 (43.8)	
There is a hierarchy in my relationship with the mental health physicians with whom I work.	Agree	62 (35.4)	26 (38.8)	19 (37.3)	8 (32.0)	9 (28.1)	0.733
	Disagree	113 (64.6)	41 (61.2)	32 (62.7)	17 (68.0)	23 (71.9)	
Psychiatrists are accessible if and when I want to consult with them.	Agree	63 (36.0)	23 (34.3)	22 (43.1)	12 (48)	6 (18.8)	0.075
	Disagree	112 (64.0)	44 (65.7)	29 (56.9)	13 (52.0)	26 (81.3)	
I am accessible if psychiatrists want to consult with me.	Agree	122 (69.7)	52 (77.6)	35 (68.6)	17 (68.0)	18 (56.3)	0.188
	Disagree	53 (30.3)	15 (22.4)	16 (31.4)	8 (32.0)	14 (43.8)	
My collaboration with my client’s psychiatrist is necessary for the care of my client.	Agree	158 (90.3)	60 (89.6)	48 (94.1)	21 (84.0)	29 (90.6)	0.567
	Disagree	17 (9.7)	7 (10.4)	3 (5.9)	4 (16.0)	3 (9.4)	
I feel respected by psychiatrists during our periods of contact regarding patient care.	Agree	104 (59.4)	50 (74.6)	27 (52.9)	15 (60.0)	12 (37.5)	0.003
	Disagree	71 (40.6)	17 (25.4)	24 (47.1)	10 (40.0)	20 (62.5)	
I think that mental health physicians should be employed in primary healthcare settings.	Agree	130 (74.3)	54 (80.6)	40 (78.4)	17 (68.0)	19 (59.4)	0.107

## Discussion

Family and primary healthcare physicians are the first point of medical contact for psychiatric patients in most situations [9]. In addition, family and primary care physicians play a vital role in providing psychiatric services at a primary care level; therefore, they need to collaborate with psychiatric professionals in order to be able to provide such services effectively [10]. The WHO has suggested integration between primary healthcare systems and mental health care providers in order to provide psychosocial healthcare to all patients more effectively [7].

In Saudi Arabia, psychiatric problems are prevalent in all ages and sectors of the community, as has been shown in previous studies [4,11-15]; however, an accurate estimate of the prevalence of psychiatric issues among the Saudi population is lacking [16]. In order to determine the quality of the current mental health services being provided at the primary healthcare level, it is necessary to assess the primary care physicians’ attitudes toward collaboration with psychiatrists.

In the present study, most of the family physicians had received training in mental health, and approximately half of them had interned in psychiatric settings. However, almost 90% agreed that collaboration with psychiatrists is necessary for the care of their patients, and approximately three-quarters agreed that mental health physicians should be employed in primary healthcare settings. This indicated the need for family physicians to collaborate with psychiatrists when caring for their patients.

Most of the physicians in our sample had received inter-professional education as part of their doctoral program and inter-professional training in a clinical training program. However, the majority of them believed that family physicians need to be better educated and trained regarding the identification of mental health problems, and more than half agreed that their current collaboration with psychiatrists was not effective in optimizing patient care. It has been documented that collaborative care between psychiatrists and primary care physicians ranges from occasional communication to close collaboration and teamwork [17].

Although the majority of the family physicians in this study were in favor of collaboration with psychiatrists when providing care to psychiatric patients, only one-third of them agreed that psychiatrists were accessible if and when they wanted to consult with them; additionally, almost one-third of them claimed that they were not accessible if psychiatrists wanted to consult with them. Therefore, implementing and improving this collaboration should be a priority for health authorities. Meeting the needs of psychiatric patients and improving healthcare services are advantages of the collaboration between family/primary healthcare physicians and psychiatrists [18].

Qureshi et al. [19] reported that the integration of primary healthcare and psychiatric services could change the referral patterns of patients from primary healthcare to psychiatry. However, in the present survey, less than half of the family physicians claimed that they referred patients with mental health disorders to a psychiatrist or consulted with a psychiatrist about their patients with mental health disorders monthly, whereas almost a third of them did this once a year or less.

In this study, 20% of the family physicians reported conducting psychotherapy and mental health consultation themselves. Across various studies, the appropriateness of family or primary healthcare physicians treating psychiatric disorders varied considerably, and most of this variation was attributed to different educational backgrounds, styles of communication, and diagnostic and therapeutic skills [20].

Among the important limitations of the present study was the inclusion of family physicians from only one city (Jeddah). Therefore, the results could be subjected to selection bias and are not applicable to family physicians from other places in the Kingdom of Saudi Arabia. In addition, data collection involved a self-administered questionnaire, with no direct observation of practice, which is also susceptible to bias. Despite these limitations, this study does have a significant value given its exploration of the need for closer collaboration between family/primary healthcare physicians and psychiatrists in order to improve the overall quality of services delivered to psychiatric patients at the primary care level.

## Conclusions

Family physicians had satisfactory attitudes toward collaboration with psychiatrists; however, this collaboration was not optimal from a practical point of view and should be improved through the provision of continuous medical education for family physicians, developed and implemented by psychiatrists. Additionally, more effective communication should be encouraged in order to deliver better care to psychiatric patients in primary care settings. A local program is recommended to ensure better training in psychiatry.

## Appendices

**Table 8:** Physicians’ agreement on collaboration with psychiatrists based on internship in a psychiatric setting

Items	Total (n=175) n (%)	Internship in a Psychiatric Setting n (%)		p-value
		Yes (n=80)	No (n=95)	
My education prepared me well for collaboration with psychiatrists.	111 (63.4)	52 (65.0)	59 (62.1)	0.692
I think family physicians need to be better educated and trained regarding the identification of mental health problems.	161 (92.0)	75 (93.8)	86 (90.5)	0.434
My current collaboration with psychiatrists is effective for optimizing client care.	82 (46.9)	41 (51.3)	41 (43.2)	0.285
I am comfortable giving feedback about a client to their psychiatrist.	124 (70.9)	59 (73.8)	65 (68.4)	0.440
There is a hierarchy in my relationship with the mental health physicians with whom I work.	62 (35.4)	33 (41.3)	29 (30.5)	0.140
Psychiatrists are accessible if and when I want to consult with them.	63 (36.0)	32 (40.0)	31 (32.6)	0.312
I am accessible if psychiatrists want to consult with me.	122 (69.7)	54 (67.5)	68 (71.6)	0.559
I think my collaboration with my client’s psychiatrist is necessary for the care of my client.	158 (90.3)	71 (88.8)	87 (91.6)	0.529
I feel respected by psychiatrists during our periods of contact regarding patient care.	104 (59.4)	49 (61.3)	55 (57.9)	0.652
I think that mental health physicians should be employed in primary healthcare settings.	130 (74.3)	58 (72.5)	72 (75.8)	0.620
